# Three Different Ways of Treating Primary Hepatocellular Carcinoma at an Early Stage: A Prospective Comparative Study

**DOI:** 10.1155/2020/7802498

**Published:** 2020-05-15

**Authors:** Di Wu, Yue Yang, Jing Chen, Huihua Cai, Yunfei Duan, Donglin Sun

**Affiliations:** Department of Hepatopancreatobiliary Surgery, Changzhou First People's Hospital, Third Affiliated Hospital of Soochow University, 185th Juqian Street, Changzhou 213001, China

## Abstract

Primary hepatocellular carcinoma (PHC) is one of the most common malignancies in clinical practice. According to the “Guidelines for Diagnosis and Treatment of Primary Liver Cancer in China,” PHC, at an early stage, can be treated by surgical resection and ablation. Surgical resection basically consists of two ways; one is open hepatectomy (OH), and the other is laparoscopic hepatectomy (LH), which is a newly developed technique associated with advantages of open surgery. Ablation, also known as percutaneous thermal ablation using radiofrequency ablation (RFA) and microwave ablation (MWA), is a minimally invasive curative treatment for hepatocellular carcinoma. This preliminary report was aimed at evaluating the postoperative outcome of the patients undergoing these three therapeutic methods, respectively. The study analyzed the data of 95 patients who underwent LH, OH, or ablation between June 2018 and June 2019 at First People's Hospital of Changzhou, Third Affiliated Hospital of Soochow University. There were 20 patients in the ablation group, 35 patients in the OH group, and 40 patients in the LH group. Among the three groups, the postoperative short-term outcome was the best in the ablation group, suggesting that it was a safe and cheap way to treat PHC at an early stage.

## 1. Introduction

Primary hepatocellular carcinoma (PHC) is the fourth most common malignant tumor and the third leading cause of tumor-related death in China [1, 2], and it is also the leading cause of death worldwide [3]. Treatment choice can be made among surgical resection, ablation, TACE (transarterial chemoembolization), systemic treatment, radiotherapy, and liver transplantation. PHC at an early stage can be treated by two ways. The main treatment is partial hepatectomy which includes two surgical procedures; one is laparoscopic hepatectomy (LH), and the other is open hepatectomy (OH). Ablation is another choice for patients with PHC at a stage of Ia and Ib [4].

Since the first laparoscopic hepatectomy (LH) was performed in 1991 by Ciria et al., laparoscopic hepatectomy has been widely performed in the world, and it has become the standard procedure for hepatectomy [5]. Many consensus guidelines for LH, such as the Louisville Statement and the Morioka Statement, standardize the indications and procedures of LH and promote the development of LH to a large extent [6, 7]. At present, LH is a good choice for liver tumors of different sizes and locations.

Local ablation is guided by imaging technology to target the tumor and kill the tumor tissue by physical or chemical methods. The characteristics of local ablation therapy are as follows: first of all, it directly targets the tumor and has the advantage of high efficiency and rapidity; secondly, the treatment range is limited to the tumor and its surrounding tissues, which has little effect on the function of the liver and the whole body, so, it can be performed repeatedly [8]. Because of its definite curative effect, especially in small liver cancer, the effect of ablation is similar to that of surgical resection, so it is considered one of the radical treatment methods for small liver cancer [9, 10].

At present, many studies have focused on the long-term efficacy of LH, OH, and ablation in liver cancer [11, 12]. Few has paid much attention to the differences of short-term outcomes including blood loss, operation time, surgical outcomes, length of hospital stay, total hospitalization expenses, and recovery of liver function.

This study mainly focused on the comparison of short-term outcomes of the three methods to further illustrate the safety and efficacy of LH, OH, and ablation in treating primary hepatocellular carcinoma at an early stage.

## 2. Materials and Methods

### 2.1. Study Design and Data Collection

This study is a prospective comparative study performed in a single center. Data were collected during the study process.

### 2.2. Patient Selection and Grouping

PHC patients admitted to the department of hepatopancreatobiliary surgery were enrolled in our study in case of meeting the criteria we made. The criteria involved the following rules: normal cardiac and pulmonary function; tolerating the risk of general anesthesia; liver function of Child-Pugh A/B; without extrahepatic metastases; no active hepatitis; without blood vessel invasion; single lesion; and the size of tumors no larger than 5 cm.

Patients who had undergone upper abdominal surgery and patients who refused laparoscopic hepatectomy or ablation were placed in the OH group. Others went to the LH group or ablation group. Patients whose tumors were located on the edge of the liver were selected into the LH group, while patients whose tumors were located in the center of the liver were selected into the ablation group.

### 2.3. Surgical Procedures

#### 2.3.1. OH Procedures

Patients were placed in supine position, and a general anesthesia was administered. Laparotomy was performed through anti-L-shaped incision or midline incision in the epigastrium. Irregular hepatectomy was performed, and the resection range included 2 cm from the edge of the tumor. Harmonic scalpel was used to separate loose tissue and liver tissue. Blood vessels and bile ducts (≥5 mm in diameter) were ligated carefully.

#### 2.3.2. LH Procedures

Patients were placed in large font position, which largely facilitates the assistant who holds the laparoscope, and a general anesthesia was administered. Intra-abdominal pressure was maintained at 12 mmHg (1 mmHg = 0.133 kPa). One 10 mm trocar was inserted above the umbilicus as an observation hole, and other four trocars were placed on the left and right sides of the abdomen as operating holes. Irregular hepatectomy was performed, and the resection range included 2 cm from the edge of the tumor. Harmonic scalpel was used to separate loose tissue and liver tissue. Hem-o-lok clips and titanium clips were used to ligate blood vessels and bile ducts (≥5 mm in diameter). Intraoperative ultrasonography was used to identify the relationships between the tumor and main blood vessels and bile ducts to avoid unnecessary injury, and it could also detect hidden metastasis in the liver which was not found during preoperative examination.

#### 2.3.3. Ablation Procedures

Surgery must be conducted after detailed ultrasound examination or reading CT to get a full evaluation of the liver tumors and to make a reasonable needle path. After general anesthesia, the operation area was disinfected and the towels were spread. Ultrasound was performed again to determine the insertion point, the insertion angle, needle placement, and the insertion protocol. We tried to choose a way which passed through the intercostal space and part of the normal liver tissue before reaching the tumor. Ablations were performed using radiofrequencies. In order to ensure the effect of ablation therapy, the ablation range should cover 0.5 cm beyond the tumor boundary. After ablation, the needle track was ablated to prevent postoperative bleeding and tumor implantation along the needle track. After that, ultrasound was performed to check if there was any possible abdominal bleeding and residual tumor.

### 2.4. Postoperative Management

All patients stayed in the ICU in the ward until their vital signs were stable usually for one day or two. Liver function test and routine blood tests were conducted at 1st, 3rd, 5th, and 7th day postoperation. CT scan was performed to detect if there was any residual fluid in the peritoneal cavity. If there was not any residual fluid in the peritoneal cavity and drainage fluid was serous and there is absence of bile leakage, the abdominal drainage tube was removed. After that, when the liver function returned to near-normal levels, the patient was discharged.

### 2.5. Statistical Analysis

Normal distributions of numerical variable data were verified with Shapiro-Wilk test. In case normal distributions were verified, numerical variable data were presented as mean and standard deviation (SD) and compared by analysis of variance (ANOVA) (*F* test). Categorical variable data were presented as number and percentages and compared by the *χ*^2^ test and Fisher's exact test [13]. SPSS software (SPSS Statistics 17.0) was used for data analysis, and a statistically significant difference was considered for a value of *P* < 0.0522 [14].

## 3. Results

### 3.1. Patient Grouping

According to the criteria mentioned above, 95 patients were included in this study. They were divided into three groups (OH group (*n* = 40), LH group (*n* = 35), and ablation group (*n* = 20)).

### 3.2. Homogeneity of Patients

The characteristics of 95 patients (OH group (*n* = 40), LH group (*n* = 35), and ablation group (*n* = 20)) included are presented in Table 1. Age, sex, hepatitis B positive rate, tumor size, alpha-fetoprotein (AFP) positive rate, abnormal prothrombin positive rate, and Child-Pugh grade were involved. There were no significant differences between these groups among these factors (*P* > 0.05).

### 3.3. Intraoperative Condition and Postoperative Recovery Situation

Intraoperative conditions of the three groups are shown in Table 2. The incision length of the LH group was significantly shorter than that of the OH group (*P* < 0.05). While in the ablation group, there was no incision at all, only one or two needle marks remained on the skin. As with operating time, the ablation group was the shortest, followed by the OH group, while the LH group was the most time-consuming (*P* < 0.05). Blood loss and blood transfusion during operation were significant less in the LH group than in the OH group (*P* < 0.05). In the ablation group, there was little bleeding or transfusion during the operation.

Postoperative recovery situation showed that the exhaust and defecation time was significantly shorter in the LH group than in the OH group (both *P* < 0.05). In the ablation group, it took an average time of approximately 1.35 d and 1.55 d to exhaust and defecate, significantly shorter than the other two groups (*P* < 0.05). The ambulation time, the extubation time, and postoperative hospital stay were the shortest in the ablation group, followed by the LH group and then the OH group (all *P* < 0.05). In the LH group, postoperative blood transfusion was less than that in the OH group (*P* < 0.05). In the ablation group, no blood transfusion was needed. As with total hospitalization expenses, the LH group was the most expensive, then comes with the OH group, and the ablation group was the cheapest (*P* < 0.05). According to our observation, surgical cost and postoperative hospital stay were the main determinants of hospitalization expenses.

### 3.4. Recovery of Liver Function

Pre-and postoperative liver function data are provided in Table 3. There was no significant difference in liver function between the three groups before operation (*P* > 0.05). Postoperatively, concentration of alanine transaminase (ALT), aspartate transaminase (AST), total bilirubin (TB), and direct bilirubin (DB) was the lowest in the ablation group at 1st day, 3rd day, 5th day, and 7th day after operation; then came the LH group and the OH group (*P* < 0.05). On the contrary, concentration of albumin rises up at the fastest speed in the ablation group and the slowest speed in the OH group, while the LH group in the middle (*P* < 0.05).

## 4. Discussion

PHC patients with liver function of Child-Pugh A/B, without extrahepatic metastases, without blood vessel invasion, single lesion, and the size of tumors no larger than 5 cm are classified as stage Ia. They can be treated by either surgical resection or ablation according to “Guidelines for Diagnosis and Treatment of Primary Liver Cancer in China” (2017 Edition). At present, some data show that laparoscopic hepatectomy can achieve the same long-term survival effect in some selective patients, but the perioperative complications are significantly reduced compared with OH [15]. In recent years, image-guided ablation plays an important role in the treatment of liver cancer, especially radiofrequency ablation and microwave ablation. Because of its advantages of small trauma, easy to conduct, effective coagulation, and inactivation of tumor, the therapeutic effect of ablation has made a breakthrough in small hepatocellular carcinoma [8, 16].

In this study, 95 patients were involved, and age, sex, hepatitis B positive rate, tumor size, alpha-fetoprotein (AFP) positive rate, abnormal prothrombin positive rate, and Child-Pugh grade were all proved to be homogeneous.

The incision length of the LH group was significantly shorter than that of the OH group, while in the ablation group, there was no incision at all; only one or two needle marks remained on the skin. Small incision means less chance of postoperative incision pain, and patients are more willing to ambulate after surgery at an early time. So ambulation time was the shortest in the ablation group followed by the LH group and OH group. As we all know, early ambulation time after operation is helpful to the recovery of intestinal peristalsis. So, the results were the same with defecation and exhaust time. As with operating time, the ablation group was the shortest, followed by the OH group, while the LH group was the most time-consuming. In fact, according to our observation, the time of ablation procedure was actually very short, which took no more than 15 min. Intraoperative ultrasound radiography-guided localization took a long time, so localization technique needs to be improved. As with the LH group, bleeding control and intraoperative exposure were more difficult, so it took a longer time in the LH group than the other groups [17]. To our surprise, blood loss and blood transfusion during operation were significantly less in the LH group than in the OH group. We suppose that it was because of the magnifying effect of the laparoscope which made the blood vessel clearer so that unnecessary damage can be avoided. Almost no blood loss was observed in the ablation group, so no blood transfusion was performed. The extubation time was the shortest in the ablation group, followed by the LH group and then the OH group. Actually, a drainage tube was not necessary in the ablation group at all. It took a longer time to remove the drainage tube in the OH group than in the LH group probably because small blood vessels were not well dealt with as the LH group. Some research revealed that less abdominal ascites, small wound surface, and reduced stress response were conducive to shorten the abdominal drainage time [3]. A total hospitalization expense of the LH group was the most expensive for its more use of newly developed devices such as LigaSure, high-definition laparoscope, and endoscopic stapler [18, 19].

The recovery of liver function in each group revealed that after surgery, the concentration of ALT, AST, TB, and DB was the highest in the OH group followed by the LH group and ablation group, and the opposite result can be seen in the concentration of ALB. The results indicated that the recovery of liver function was the fastest in the ablation group, and then came the LH group and OH group. Studies have reported that liver injury in LH is milder which is conducive to the recovery of liver function in patients. Without hepatectomy, liver injury was the mildest in the ablation group, so the liver function came back faster than the other groups. Early exhaust and defecation time can lead to early feeding, which can not only provide the body with nutrition to produce ALB but also promote blood circulation in hepatic portal veins to facilitate the intestinal absorption of nutrients which can directly enter into the liver and participate in its repair [20]. When faced with trauma or stress, the liver would enhance albumin synthesis, but when damage is severe or exceeds liver compensatory capacity, it may result in insufficient albumin synthesis [21].

In conclusion, ablation is a good choice for PHC patients. There are advantages in both price and short-term effects, and we strongly recommend it as a routine method for patients with poor liver function because it has minor impact on liver function. LH has better short-term outcomes than the OH procedure with the advantages of less injury and quick recovery although it is slightly more expensive than OH.

However, there is insufficiency in our previous study. For the sake of time, the data is not complete for its lack of long-term follow-up data. It may indicate the long-term survival rates of the patients, which is another important point in making a decision of which method to choose.

## 5. Conclusions

Ablation is a safe and cheap way to treat PHC at an early stage for its wonderful performance in the postoperative short-term outcome.

## Figures and Tables

**Table 1:** Homogeneity of patients.

Parameters	OH	LH	Ablation	*F* test/square test	*P*
Age (years)	58.6 ± 10.52	61.8 ± 8.51	61.6 ± 6.67	1.37	0.259
Sex (%)					
Female	7	5	3	0.157	0.924539
Male	33	30	17		
HBsAg (%)					
Positive	32	28	15	0.2375	0.88803
Negative	8	7	5		
Tumor size (cm)	3.54 ± 0.65	3.56 ± 0.68	3.50 ± 0.54	0.345	0.709
AFP (%)					
Positive	25	19	11	0.6038	0.739412
Negative	15	16	9		
Abnormal prothrombin (%)					
Positive	26	17	12	2.1128	0.347705
Negative	14	18	8		
Child-Pugh grade (%)					
A	35	29	17	0.3216	0.85462
B	5	6	3		

AFP: positive > 20 *μ*g/L and negative ≤ 20 *μ*g/L; abnormal prothrombin: positive > 40 AU/L and negative ≤ 40 AU/L.

**Table 2:** Intraoperative condition and postoperative recovery situation.

Parameters	OH	LH	Ablation	*F*	*P*
Incision length (cm)	29.98 ± 3.20	4.8 ± 0.80	0	1861.746	0.000
Operating time (min)	186.5 ± 37.11	207.71 ± 27.45	70.25 ± 11.53	145.935	0.00
Intraoperative bleeding (mL)	305.75 ± 149.85	288 ± 111.06	3 ± 00.29	48.745	0.00
Intraoperative blood transfusion (mL)	126.25 ± 175.04	54.29 ± 119.66	0	6.353	0.003
Exhaust time (days)	3.3 ± 0.77	2.63 ± 0.49	1.35 ± 0.49	66.417	0.000
Defecation time (days)	3.68 ± 0.66	2.94 ± 0.42	1.55 ± 0.51	100.329	0.000
Ambulation time (days)	2.78 ± 0.66	1.86 ± 0.55	1.2 ± 0.41	55.1	0.000
Extubation time (days)	6.85 ± 1.08	5.49 ± 0.66	0	495.264	0.000
Postoperative hospital stay (days)	8.4 ± 1.01	7.46 ± 0.78	5.5 ± 0.69	74.456	0.000
Total hospitalization expenses (RHB, yuan)	48641.95 ± 3073.82	53958.11 ± 5549.77	48364.71 ± 3745.31	17.718	0.000
Postoperative blood transfusion (mL)	195 ± 170.90	140 ± 155.68	0	10.513	0.000

**Table 3:** Recovery of liver function.

Parameters	OH group	LH group	Ablation group	*F*	*P*
ALT (U/L)					
Preoperation	51.53 ± 12.83	50.94 ± 12.25	50.10 ± 4.73	0.105	0.900
Postoperation					
1st day	535.75 ± 82.52	498.86 ± 81.30	433 ± 81.89	10.494	0.000
3rd day	474.33 ± 76.35	434.37 ± 75.64	381.55 ± 75.40	10.119	0.000
5th day	403.7 ± 69.23	319.86 ± 80.61	144.8 ± 26.22	97.693	0.000
7th day	224.73 ± 50.18	174.49 ± 49.69		18.885	0.000
AST (U/L)					
Preoperation	37.2 ± 21.02	36.2 ± 33.33	31.45 ± 7.37	0.377	0.687
Postoperation					
1st day	446.13 ± 71.19	367.8 ± 57.33	266.65 ± 47.77	56.987	0.000
3rd day	370.83 ± 55.43	263.69 ± 40.18	147.1 ± 24.77	170.506	0.000
5th day	262.55 ± 39.09	156.4 ± 27.19	89.75 ± 22.62	219.292	0.000
7th day	158.5 ± 26.77	95.17 ± 22.57		120.728	0.000
Albumin (g/L)					
Preoperation	43.03 ± 5.31	41.71 ± 4.46	40.6 ± 3.45	1.929	0.151
Postoperation					
1st day	23.5 ± 2.44	26.54 ± 2.19	29.5 ± 2.26	47.156	0.000
3rd day	24.9 ± 1.69	28.46 ± 1.79	31.7 ± 1.42	116.269	0.000
5th day	26.83 ± 1.71	29.91 ± 1.31	34.4 ± 1.23	176.424	0.000
7th day	30.63 ± 1.75	32.71 ± 1.71		27.221	0.000
Total bilirubin (*μ*mol/L)					
Preoperation	19.40 ± 4.06	19.00 ± 3.98	18.68 ± 3.57	0.243	0.785
Postoperation					
1st day	35.37 ± 8.18	29.46 ± 2.84	23.7 ± 1.22	30.161	0.000
3rd day	31.25 ± 6.75	26.49 ± 3.28	22.3 ± 2.43	11.299	0.000
5th day	28.48 ± 3.82	23.97 ± 2.74	20.05 ± 0.99	55.051	0.000
7th day	25.43 ± 3.76	21.91 ± 2.19			
Direct bilirubin (*μ*mol/L)					
Preoperation	6.51 ± 1.03	6.71 ± 0.93	7.04 ± 1.19	1.748	0.180
Postoperation					
1st day	14.55 ± 3.09	12.53 ± 2.17	10.40 ± 1.35	19.394	0.000
3rd day	12.70 ± 1.63	10.41 ± 1.35	7.92 ± 1.45	70.601	0.000
5th day	10.65 ± 1.42	8.07 ± 1.45	6.63 ± 0.48	74.431	0.000
7th day	8.24 ± 1.34	6.67 ± 0.39		44.764	0.000

## Data Availability

The data sets used and/or analyzed during the current study are available from the corresponding author on reasonable request.
